# Mesenchymal Stromal Cell Therapy Restores Intestinal Integrity and Attentuates Inflammation in a Preterm Piglet Model of Necrotizing Enterocolitis

**DOI:** 10.21203/rs.3.rs-7285196/v1

**Published:** 2025-08-18

**Authors:** Jasmine Lee, Sharon Joseph, Krishna Manohar, Fikir Mesfin, Chelsea Hunter, John Brokaw, W. Chris Shelley, Jianyun Liu, Robyn McCain, Christa J. Crain, Timothy Lescun, Troy A. Markel

**Affiliations:** Indiana University School of Medicine; Indiana University School of Medicine; Indiana University School of Medicine; Indiana University School of Medicine; Indiana University School of Medicine; Indiana University School of Medicine; Indiana University School of Medicine; Indiana University School of Medicine; Purdue University; Purdue University; Purdue University; Indiana University School of Medicine

**Keywords:** Mesenchymal Stromal Cells, Necrotizing Enterocolitis, Preterm Piglet, Stem Cell Therapy, Inflammation

## Abstract

**Purpose:**

Necrotizing enterocolitis (NEC) is a life-threatening gastrointestinal disease of prematurity characterized by inflammation, necrosis, and high morbidity. Current therapies are limited, necessitating the development of novel treatments. Mesenchymal stromal cells (MSCs) have shown promise in murine NEC models. Given the anatomical and physiological similarities between premature piglets and human infants, we employed a preterm piglet model to evaluate MSC efficacy. We hypothesized that intraperitoneal MSC administration would reduce intestinal injury in NEC.

**Methods:**

Preterm piglets were delivered via cesarean section. NEC was induced on day 3 through hypertonic enteral feeding. MSCs were administered intraperitoneally at low, medium, or high doses. Piglets were monitored and euthanized based on clinical criteria. Clinical scores, weight change, gross and histologic intestinal injuries were assessed. Cytokine levels in serum and ileum were quantified via ELISA, and intestinal tissue was analyzed by RNA sequencing. Statistical significance was set at p < 0.05.

**Results:**

Medium-dose MSCs significantly improved clinical scores and reduced both gross and histologic intestinal injury (p < 0.05). A corresponding decrease in pro-inflammatory cytokines was observed.

**Conclusion:**

This is the first study to demonstrate therapeutic benefit of MSCs in a preterm piglet NEC model, supporting their potential use in translational NEC therapies.

## 1. INTRODUCTION

Necrotizing enterocolitis (NEC) is a devastating gastrointestinal condition commonly affecting premature infants. The overall mortality for NEC is 20–30%[1–3] and the mortality in premature infants is as high as 50%[4]. It is characterized by intestinal inflammation and necrosis, leading to significant morbidity and mortality[4–7]. Severe NEC can present with full thickness intestinal destruction, which can lead to intestinal perforation, peritonitis, and sepsis. The etiology of NEC is multifactorial, with prematurity and immaturity of the gastrointestinal tract being primary risk factors, in addition to factors such as formula feeds, gut dysbiosis, infection, or intestinal hypoperfusion[8–10]. Despite extensive research, the incidence and mortality of NEC are largely unchanged, and it remains the most common cause of gastrointestinal mortality among newborns[1].

Current treatment options for NEC are limited, highlighting the need for innovative therapeutic strategies. Mesenchymal stromal cell (MSC) therapy has emerged as a promising approach due to their potential to modulate inflammation and promote tissue repair[11]. MSC therapy has shown promise in many diseases of premature infants including bronchopulmonary dysplasia, brain injury, and retinopathy of prematurity[12–15]. A growing number of preclinical studies have investigated the therapeutic role of MSCs in experimental NEC and other models of intestinal ischemia[16–23]. These studies utilize either a mouse or rat model, which, while valuable, may not best replicate the complexities of human neonatal physiology. Therefore, exploring alternate models, such as piglets, may provide more relevant insight.

In this study, we utilized a premature piglet model to investigate the efficacy of MSC therapy in mitigating the intestinal injury associated with NEC. Premature piglets are an ideal model for this research because their size and gastrointestinal development are similar to human neonates[24, 25]. This allows for the assessment of different concentrations of MSCs on the impact of intestinal injury. Premature piglets also have shown spontaneous development of NEC with early initiation of infant formula feeds[26, 27]. This similarity not only enhances the translational potential of our findings but also provides a more accurate representation of the human physiology. We hypothesized that the administration of mesenchymal stromal cells would significantly reduce intestinal injury in this premature porcine model of NEC.

## 2. METHODS

### Premature piglet model

All experiments were conducted according to institutional regulations and approved by the Purdue University IACUC (IACUC #2111002218). Sows were transported to Purdue Large Animal Hospital on the morning of surgery after fasting overnight. Sows were sedated prior to cesarean delivery using Telazol (tiletamine/zolazepam) and xylazine. Premature piglets were delivered via cesarean section at 103 days gestation, prior to the piglets reaching term at 115 gestational days. Piglets were resuscitated with light chest stimulation, nasopharyngeal suctioning, supplemental oxygen, and 1–2 drops of atipamezole (5mg/mL) to reverse maternal anesthetic effects. Control piglets were sacrificed, and tissue was harvested within 4 hours of birth to establish baseline characteristics of preterm piglets. After resuscitation, radiofrequency temperature probes (2.1mm × 13mm, UID Temperature Programmable Microchip UCT-2112) were inserted subcutaneously, and central line catheters (Culex^®^ Rat femoral vein catheter, Model CX-2021S) were inserted into the jugular vein via a cutdown method. Piglets were placed in heated individual polystyrene incubator/nebulizer boxes equipped with water jacketed heating blankets. Homeostatic temperature was maintained over the course of the 5-day experimental study as established in the previously described model[26, 28–30].

### NEC protocol

NEC was induced in piglets with total parenteral nutrition (TPN) starting on day 1 and hypertonic oral feeding via orogastric tube on day 3. TPN was initiated immediately after central venous access was achieved at a rate of 5ml/kg/hr and continued until the end of the experiment. Parenteral nutrition provided (kg/day) 410 kJ energy, 12.5 g dextrose, 6.5 g amino acids, and 2.5 g fat (Intralipid 20%). Maternal plasma was administered intravenously at 4, 5, and 7 ml/kg at 6, 12, and 22 h after birth, respectively, to provide passive immunity. Orogastric feeding tubes (5-Fr, NEOMED) were inserted prior to the initiation of gastric feeds. Position was confirmed with auscultation prior to the initiation of the first feed and if there were concerns for tube dislodgement. On day 3, enteral feeding was initiated at 15 ml/kg every 3 h for 42 h. Caloric nutrient content of the enteric formula is provided in *Supplementary Table 1*. Piglets (n = 6–8 per group) were monitored for the duration of the experiment with hourly pulse oximetry, temperature, and scheduled evaluations of clinical sickness. Clinical sickness scores were assigned using a standardized scoring rubric (see *Supplementary Table 2*). Weight change over the course of the experiment was documented. Piglets were euthanized via cardiac injection of sodium pentobarbital 390mg/ml dosed at 0.22ml/kg while under isoflurane anesthesia. All piglets who survived the length of the study were euthanized on day 5. At the time of euthanasia, serum was isolated from collected blood. Tissue from the terminal ileum were harvested uniformly and segments were placed in histology processing/embedding cassettes.

### Cell Culture

Human induced pluripotent MSCs (iPMSC) were acquired from Cynata Therapeutics (Cremome, Australia). These MSCs were cultured in a serum free expansion medium consisting of 47.5% Human Endothelial Serum-Free Media (Thermofisher, Waltham, MA), 51.5% Stemline II Hematopoietic Stem Cell Expansion Media (Sigma-Aldrich, St. Louis, MO), 2 mM Glutamax (Thermo Fisher, Waltham, MA), 0.01 ng/mL Fibroblast Growth Factor (FGF-2) (R&D Systems, Minneapolis, MN), and 0.1 μM 1-thioglycerol. iPMSCs were cultured on fibronectin/collagen (Thermofisher, Waltham, MA /Sigma-Aldrich, St. Louis, MO) coated three-tiered 525 cm^2^ flasks. Cells were initially seeded at a density of 5,000 cells/ cm2 and passaged every 3–4 days. After reaching 80–85% confluency, iPMSCs were lifted using StemPro Accutase Cell Dissociation reagent (Thermofisher, Waltham, MA) at 0.5X and the reaction was stopped using deactivation media consisting of 4% Human Serum Albumin (25%, filtered, Nova Biologics, Oceanside, CA) and 0.5 mM EDTA in DPBS. The cells were then resuspended in 1.2mL of PBS, counted with a hemocytometer, and administered to piglets intraperitoneally in doses of 500,000 cells/kg (denoted as low-dose, or LD), 1 million cells/kg (denoted as mid-dose, or MD), or 10 million cells/kg (denoted as high-dose, or HD) using 1 mL syringes with a 21G 1.5” needle on day 3 of the experiment. Untreated piglets (NEC group) were injected with PBS vehicle.

### Clinical sickness score and Macroscopic intestinal injury

Each piglet received a clinical sickness score prior to euthanasia (based on presence of diarrhea, bloody stool, and degree of abdominal distension). During necropsy, gross intestinal injury score was blindly calculated for each piglet based on the gross findings of the bowel. Previously described gross injury scoring[31] was used to assign scores to piglets.

### Histologic intestinal injury

Histology cassettes were placed in 10% formalin and exchanged for 70% ethanol after 24–48 hours of fixation. These were embedded in paraffin, sectioned, and stained with hematoxylin-eosin. A ZEISS Axioscan was used to scan all slides. Each slide was evaluated at 10x magnification, and ten randomized photos of representative areas of the bowel were obtained. These photos were randomized and blindly scored by two independent graders. A scoring rubric previously described by *Ragan et al*[31] was used by each grader to score the tissues.

### Cytokine profiling by ELISA

Serum and terminal ileum samples were collected at necropsy and stored at − 80°C until analysis. Concentrations of pro-inflammatory cytokines (IL-6, IL-17A, and TNF-α) were quantified using commercially available porcine-specific ELISA kits (R&D Systems, Minneapolis, MN), following the manufacturers’ protocols. Cytokine levels in serum were reported as total picograms (pg) per mL sample. For terminal ileum, tissues were homogenized in lysis buffer (Buffer RLT, Qiagen), and cytokine concentrations were normalized to total protein content and expressed as pg cytokine/ng protein. All samples were run in duplicate, and standard curves were generated for each assay. Cytokine analysis was limited to the control, NEC, and medium-dose MSC (NEC + MD) groups.

### Transcriptomic Analysis

Terminal ileal tissue was harvested from neonatal piglets (*Sus scrofa*) across three experimental groups:(1) Control, (2) NEC, and (3) NEC treated with the optimal mesenchymal stromal cell dose (NEC + MD). Total RNA was extracted using the RNeasy Mini Kit (Qiagen), following the manufacturer’s protocol. RNA integrity and concentration were assessed using the Agilent TapeStation system (Agilent Technologies, Santa Clara, CA). Samples selected for sequencing had RNA Integrity Numbers ranging from 3.7 to 8.3. Given the variability in RNA quality, a total RNA sequencing protocol was employed, which has high sensitivity to partially degraded RNA.

Library preparation was performed at the Center for Medical Genomics at Indiana University School of Medicine using the Illumina Total RNA-Seq protocol, which includes ribosomal RNA depletion and strand-specific paired-end library construction. Sequencing was carried out using the Illumina NovaSeq X Plus platform with 100 bp paired-end reads and NovaSeq X chemistry.

Raw reads underwent quality control using FASTQC, and low-quality reads, including those mapped to multiple locations, were excluded. Reads were aligned to the *Sus scrofa* reference genome using STAR[32], and genomic distribution across annotated regions was assessed using bamUtils[33]. Gene-level expression quantification was performed using featureCounts[34].

Differential gene expression analysis was conducted using edgeR[35] applying negative binomial generalized linear models with likelihood ratio testing. Comparisons included NEC vs Control, NEC vs NEC + MD, and Control vs NEC + MD.

Smear plots were generated for each pairwise comparison to display log_2_-fold change versus log_2_-counts per million, highlighting significantly differentially expressed genes. Additionally, a heatmap of 15 inflammation- and injury-associated genes was constructed using the GraphPad Prism software, with expression values normalized and log-transformed to visualize relative gene expression across groups.

### Statistics

Statistical significance between the groups was analyzed using GraphPad Prism software. Statistical analysis was performed using one-way ANOVA with Sidak’s post hoc test for normally distributed data, or Kruskal-Wallis test with Dunn’s post hoc correction for nonparametric data, to compare control, NEC, and MSC-treated groups. A p-value < 0.05 was considered statistically significant.

## 3. RESULTS

Three pregnant sows delivered 40 premature piglets. Each piglet was randomized and weight-matched into each group. The final groups were comprised of 9 control piglets, 7 NEC piglets treated with PBS, 8 LD-treated piglets, 8 MD-treated piglets, and 8 HD-treated piglets.

**Figure 2:**
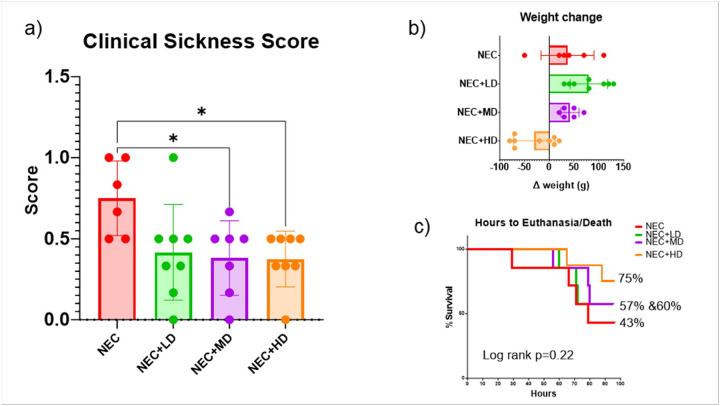
Clinical Sickness Score, Weight Gain/Loss, Survival. (a) Clinical sickness scores were significantly reduced in animals treated with mid-dose (NEC+MD) and high-dose (NEC+HD) MSCs compared to NEC animals (p=0.0447 and p=0.00335, respectively). NEC+LD animals also trended toward improvement. Asterisks indicate statistically significant differences. (b) Weight change from birth to euthanasia is shown for each group. MSC-treated animals showed less weight loss compared to untreated NEC controls, with the greatest preservation of weight observed in the NEC+HD group. (c) Kaplan–Meier survival analysis showing percentage survival over time for NEC animals with or without MSC treatment. Although a higher proportion of animals survived in the NEC+HD group (75%) compared to NEC (43%), the differences did not reach statistical significance (Log-rank p = 0.22). Final survival percentages are listed beside each group

The effect of MSCs on clinical sickness at the end of the experiment is seen in Fig. 2a. NEC piglets had significantly higher clinical sickness scores (0.75 ± 0.13) compared to both medium-dose (NEC + MD,0.38 ± 0.13) and high-dose (NEC + HD, 0.38 ± 0.13) MSC-treated groups. These differences were statistically significant (NEC vs. MD: p = 0.0447, 95% CI: 0.0069 to 0.7313; NEC vs. HD: p = 0.0335, 95% CI: 0.0234 to 0.7266). The low-dose group (NEC + LD) showed a non-significant reduction in clinical score (0.42 ± 0.13) compared to NEC (p = 0.0677, 95% CI: − 0.0183 to 0.6850). No significant differences were found among the MSC treatment groups.

Average weight change over the 5-day study period varied across groups as seen in Fig. 2b. NEC piglets gained an average of 36.7 ± 21.9 g, while MSC-treated groups showed a broader range of responses. NEC + LD and NEC + MD piglets gained 87.1 ± 13.0 g and 38.3 ± 7.5 g, respectively. In contrast, NEC + HD piglets experienced a mean weight loss of − 24.4 ± 12.8 g. Although NEC + HD animals showed a trend toward reduced weight gain compared to NEC, this difference did not reach statistical significance (p = 0.0567).

Survival over the 5-day study period varied among groups (Fig. 2c). NEC piglets had the lowest mean survival rate (42.9%), while survival improved in the NEC + LD (57.1%) and NEC + MD (60.7%) groups. The NEC + HD group exhibited the highest survival (75.0%). However, these differences were not statistically significant (log-rank test, p = 0.22).

**Figure 3:**
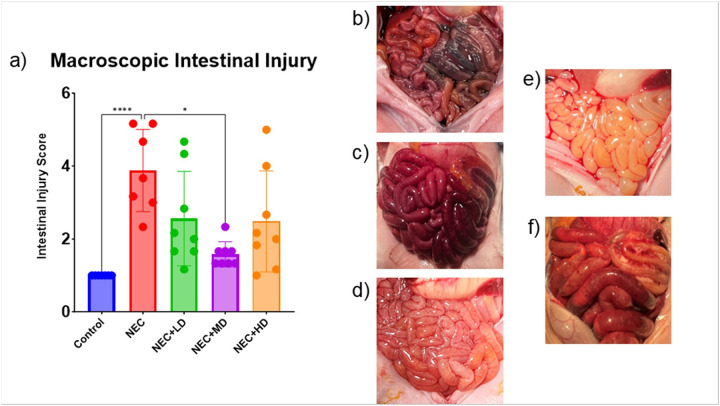
Macroscopic Intestinal Injury. (a) Macroscopic intestinal injury scores were significantly elevated in NEC animals compared to controls (p <0.0001). Treatment with mid-dose MSCs (NEC+MD) significantly reduced injury scores compared to NEC animals (p = 0.0431), while low-dose (NEC+LD) and high-dose (NEC+HD) groups showed similar trends but did not reach statistical significance. (b-f) Representative gross intestinal images from each experimental group. (b) Control animal with normal bowel appearance and meconium in colon. (c) NEC animal with severe distension, necrosis, and discoloration. (d) NEC+LD with moderate edema and focal discoloration. (e) NEC+MD with mild edema and preserved coloration. (f) NEC+HD with moderate distension and severe congestion and inflammation

NEC piglets had significantly higher gross intestinal injury scores (4.43 ± 0.20) compared to controls(1.00 ± 0.00, p < 0.0001). Among MSC-treated groups, only the medium-dose group (NEC + MD) showed a significant reduction in injury compared to NEC (1.75 ± 0.16 vs. 4.43 ± 0.20, p = 0.0431). Neither the low-dose (2.57 ± 0.20) nor high-dose (2.50 ± 0.33) MSC groups differed significantly from NEC. Representative images of intestinal injury are shown in Fig. 3B–F.

**Figure 4:**
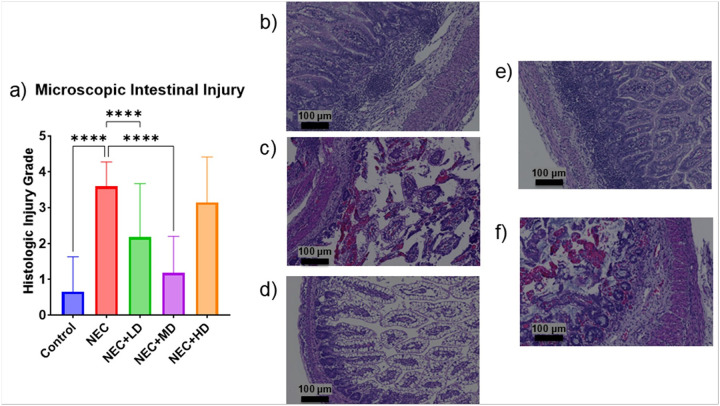
Microscopic Intestinal Injury. (a) Microscopic intestinal injury grades were significantly increased in NEC animals compared to controls (p = <0.0001). Treatment with low- and mid-dose MSCs significantly reduced histologic injury compared to NEC animals (both p<0.0001). No significant difference was observed in the NEC+HD group. (b-f) Representative hematoxylin and eosin (H&E) stained sections at 10× magnification (scale bars = 100 μm). (b) Control tissue with normal villous architecture and no inflammation. (c) NEC tissue showing marked epithelial sloughing, inflammation, and villous blunting. (d) NEC+LD showing partial preservation of architecture with reduced inflammation. (e) NEC+MD demonstrating near-normal mucosal structure. (f) NEC+HD with mild to moderate mucosal damage

The average macroscopic intestinal injury score of NEC piglets was significantly higher than the average injury score of the control group (p < 0.0001). The only MSC group showing a significant decrease from the NEC group was the medium dose group (p = 0.0431) as seen in Fig. 3. The average microscopic intestinal injury score of NEC piglets was also significantly higher than the average injury score of the control group (p < 0.0001). Both the low and medium dose showed a significant decrease in microscopic injury score compared to the NEC group (p < 0.0001 and p < 0.0001). Only the medium dose group showed no significant difference from the control group (p = 0.3027) as seen in Fig. 4.

**Figure 5:**
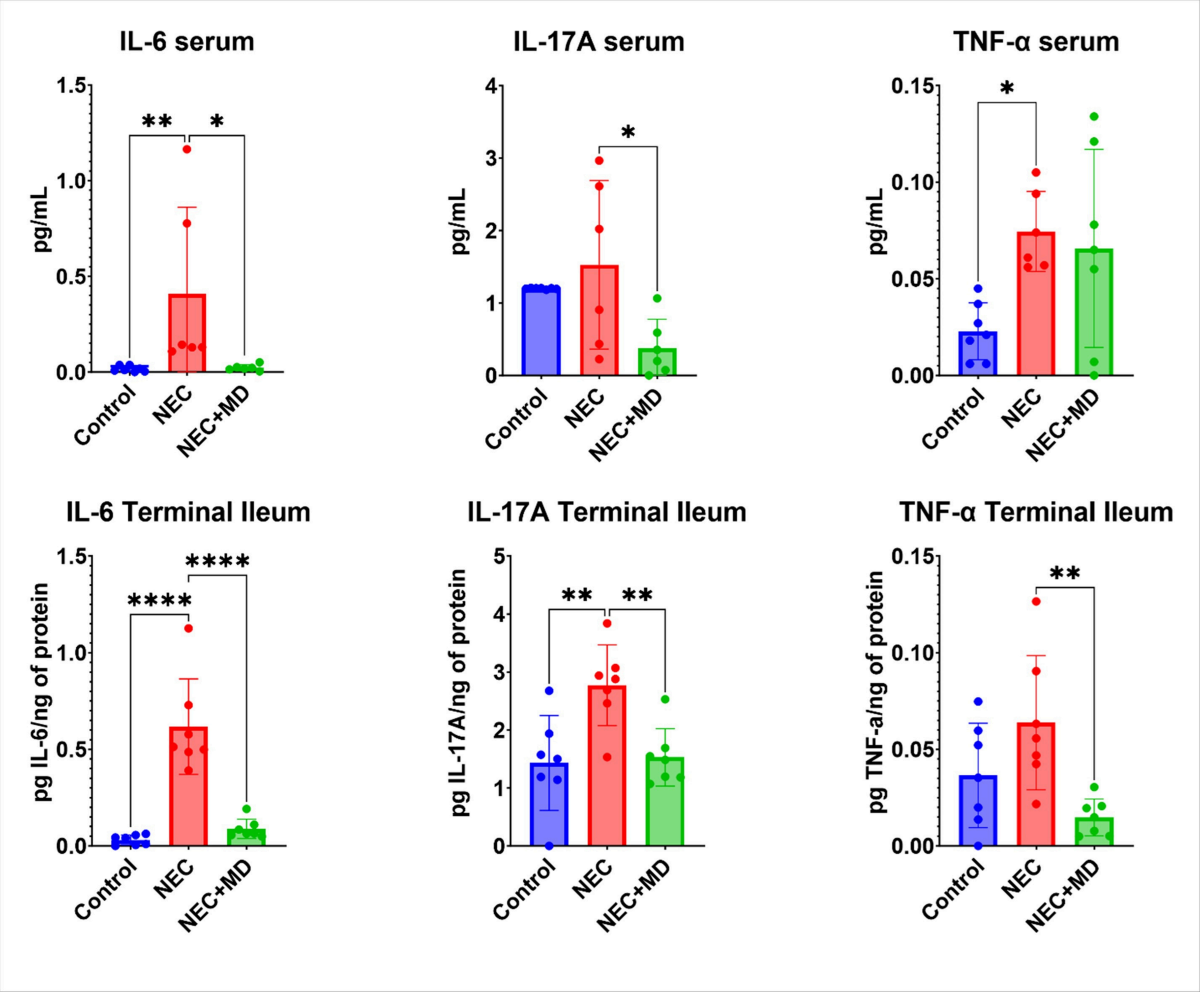
Serum and Intestinal Cytokine Profile. Pro- inflammatory cytokine levels were measured in serum (top row) and terminal ileum tissue (bottom row) from control, NEC, and NEC+MSC mid-dose (NEC+MD) animals. Serum values are shown in pg/mL, and tissue levels are normalized to total protein (pg/ng). Asterisk on graph indicated significant relationships. Significant relationships: Top row – Serum cytokines: IL-6: NEC vs. Control (p = 0.0026), NEC+MD vs. NEC (p = 0.0286); IL-17A: NEC+MD vs. NEC (p=0.0271); TNF-α: NEC vs. Control (p = 0.0338). Bottom row – Terminal ileum cytokines: IL-6: NEC vs. Control (p <0.0001), NEC+MD vs. NEC (p<0.0001); IL-17A: NEC vs. Control (p = 0.0047), NEC+MD vs. NEC (p = 0.0084); TNF-α: NEC+MD vs. NEC (p = 0.0064)

Cytokine profiling using ELISA results are displayed in Fig. 5. In the serum, the NEC group showed a significant increase from the control group in IL-6 and TNF-α(p = 0.0026 and p = 0.0338) and the medium dose MSC cell group showed a significant decrease from the NEC group in IL-6 and IL-17A (p = 0.0286 and p = 0.0271). In the terminal ileum, the NEC group was significantly higher than controls in IL-6 and IL-17A (p < 0.0001 and p = 0.0047) and the medium dose MSC group showed significant decrease from the NEC group in IL-6, IL-17A, and TNF-α (p < 0.0001, p = 0.0084, and p = 0.0064).

**Figure 6:**
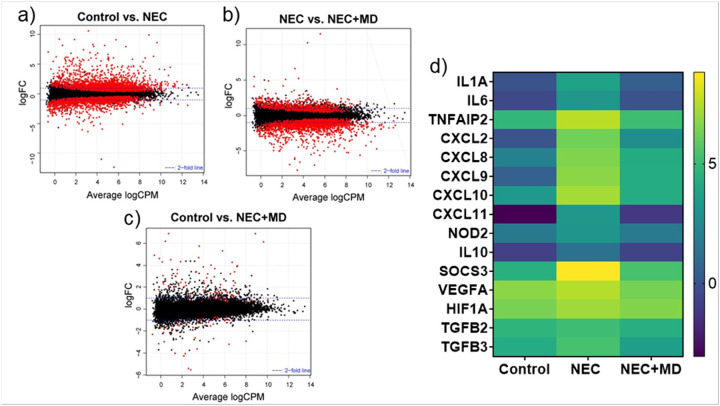
Transcriptomic Analysis of Piglet Intestinal Tissues. (a–c) Smear plots generated from RNA sequencing data showing differentially expressed genes (DEGs) across experimental groups. Each point represents an individual gene; red dots indicate genes with a false discovery rate (FDR) < 0.05. (a) Control vs. NEC. (b) NEC vs. NEC+MD. (c) Control vs. NEC+MD. (d) Heatmap of selected inflammation- and injury-associated genes. Expression values are log-transformed, normalized counts. Genes were selected based on having significantly increased expression in NEC and reduction following MSC treatment. Cytokines: IL1A, IL6, and IL10. Chemokines: CXCL2, CXCL8 (also known as IL-8), CXCL9, CXCL10, and CXCL11. Immune signaling regulators: TNFAIP2, SOCS3, and NOD2. Hypoxia and growth factor-related genes: VEGFA, HIF1A, TGFB2, and TGFB3

RNA sequencing of the piglet intestines revealed widespread transcriptional changes between the experiment groups. A total of 16621 genes passed quality filtering and were included in the differential expression analysis. In the Control vs NEC comparison, 4039 genes were significantly differentially expressed (p < 0.05), including 2375 upregulated and 1664 downregulated transcripts. In the NEC vs NEC + MD comparison, 2614 genes were significantly expressed, including 736 upregulated and 1878 downregulated transcripts. Finally, in the Control vs. NEC + MD comparison, 130 genes were significantly differentially expressed, including 85 upregulated and 45 downregulated transcripts. These results are represented in smear plots shown in Fig. 6a–c. Figure 6d shows a heatmap of 15 inflammation- and injury-associated genes from this data which demonstrate significant upregulation of expression in NEC (p < 0.05) and significant attenuation with MSC treatment.

## 4. DISCUSSION

NEC is a complex, multifactorial disease primarily influenced by hypoxic-ischemic injury to the gastrointestinal tract[3, 5, 8, 9, 36]. To the best of our knowledge, this is the first study to investigate the therapeutic use of human MSCs in a premature piglet model of NEC. Studies have shown that local transplantation of mesenchymal stromal cells effectively reduces pathological damage and preserves intestinal barrier function in models of NEC and intestinal ischemia-reperfusion injury[23, 37, 38]. These previous studies also noted that the administration of MSCs reduced enteric bacterial translocation – a hallmark characteristic that is commonly dysregulated in NEC. Additionally, human MSCs produce growth factors and increase viability and proliferative capacity of tissue after hypoxic injury[39, 40]. They even reduce apoptosis after hypoxic injury in fetal intestinal cells[40]. The literature suggests that the regenerative effects of MSCs help mitigate histopathological damage and protect the intestines from severe injury.

Our study has indicated that MSCs show significant improvement in clinical sickness in a premature piglet NEC model. This suggests that MSCs inherently protect against the multisystem derangements that NEC can impose. Our data demonstrated that MSCs administered at a dose of 1 million cells/kg (medium dose) was the only dose to show significantly improved macroscopic and microscopic intestinal injury when compared to untreated piglets. Treatment with the medium dose resulted in a significant reduction in 2 of the 3 assessed pro-inflammatory cytokines in the serum and all 3 assessed pro-inflammatory cytokines in the terminal ileum when compared to untreated NEC samples. In assessment of all 3 groups of serum and terminal ileal tissue, the MSC-treated group had no significant difference from the control group. These findings suggest a modest systemic response and a more pronounced local inflammatory response to the NEC condition which was mitigated with MSC therapy as well.

RNA sequencing analysis revealed extensive transcriptional disruption in the setting of NEC. As shown in Fig. 6A, over 2,000 genes were significantly upregulated in NEC samples compared to controls, reflecting a robust gene expression response. Following MSC treatment, more than 1,800 genes were significantly downregulated compared to NEC samples (Fig. 6B), suggesting that MSC therapy broadly reverses NEC-induced transcriptional activation. Notably, when comparing MSC-treated NEC samples to healthy controls (Fig. 6C), fewer than 150 genes remained significantly differentially expressed, indicating that MSC treatment restores the intestinal transcriptome to a profile closely resembling that of uninjured tissue.

To explore the molecular mechanisms underlying MSC therapy, we selected a panel of 15 inflammation- and injury-related genes from the data that were significantly upregulated in NEC and significantly downregulated following MSC treatment. Visualized in the heatmap (Fig. 6D), these genes include key cytokines, chemokines, and immune signaling regulators that may contribute to NEC pathogenesis and are responsive to MSC intervention. Their expression patterns provide insight into the signaling pathways modulated by MSCs and support their role in dampening intestinal inflammation at the transcriptomic level. This panel will guide future studies in our lab aimed at dissecting the pathways through which these gene products mediate MSC-driven protection.

While our findings highlight the potential therapeutic benefit of MSC treatment in an experimental NEC model, the precise mechanisms underlying this protection remain incompletely understood. Increasing evidence suggests that MSCs act primarily through paracrine signaling rather than direct engraftment[41]. These paracrine factors include a diverse array of cytokines, growth factors, and bioactive molecules that modulate inflammation, enhance tissue repair, and promote angiogenesis[41]. Our lab has identified hydrogen sulfide (H_2_S) as a key paracrine mediator released by MSCs[42–44]. H_2_S contributes to improved mesenteric perfusion via vasodilatory effect and modulates the host inflammatory response[45]. We have further shown that these effects are mediated in part through endothelial nitric oxide synthase (eNOS)- dependent pathways[46]. Together, these findings support a multifaceted, paracrine-driven mechanism by which MSCs confer protection in NEC.

An important distinction in our study is that high dose MSCs did not result in a significant reduction in intestinal injury, either microscopically or macroscopically, compared to NEC piglets. Previous preclinical studies in other disease processes have reported that excessive MSC dosing can lead to microvascular occlusion and is associated with an increased risk of thrombosis and aberrant inflammatory responses [47–50], all of which may counteract the beneficial paracrine effects of MSCs. Our data supports this concept that MSC-mediated tissue repair is not purely dose-dependent, and that overdosing may disrupt the delicate host-MSC interactions required for effective tissue healing.

Questions remain about the timing of MSC administration, and if the timing of MSC delivery relative to the onset of NEC symptoms affects outcomes. In the current literature, there is considerable variability in study designs and administration time of MSCs[51], underscoring the need for standardized protocols to determine optimal timing. The success of this study, which stands as the first of its kind as a large animal model of NEC, involved administering MSCs on day three of life. These results could serve as a crucial foundation for future investigations focused on determining the most optimal timing for MSC treatment. Furthermore, our study focused primarily on short-term outcomes, highlighting the necessity for future research to evaluate the long-term effects of MSC treatment on gut health and overall development in premature models. The long-term safety and efficacy of MSC therapy, particularly in clinical settings, remain to be fully elucidated. Key concerns include the potential for immune rejection, tumor formation, or unintended differentiation in neonates, especially considering their vulnerable physiological state[52]. These concerns are driving the investigations into the mechanism by which MSCs exert their effects in NEC, with the goal of developing safer therapies which could target these same pathways.

Despite the promising results observed in our piglet model of MSC therapy in NEC, several limitations must be acknowledged. First, the inherent differences between the piglet model and human physiology may impact the translatability of our findings to clinical settings. While piglets offer a closer approximation to human gastrointestinal development, they may not fully replicate the complexities of NEC in neonates. Another limitation to note is that our control group were piglets that were euthanized on their first day of life, whereas the NEC piglets and MSC-treated groups that made it to the end of the experiment were euthanized on day 5. This age discrepancy between the piglets could affect the results seen in our study, however, it was not feasible to maintain a cohort of premature piglets treated with colostrum because the cesarean section was a terminal procedure for the sow. Additionally, as previously mentioned, our study was limited by a relatively short follow-up period, which restricts our understanding of the long-term effects and potential complications associated with MSC therapy. Lastly, factors such as housing conditions, nutrition, and stress levels in the piglets could have introduced variability between litters that may have influenced the experimental results.

## 5. CONCLUSION

This study is the first to demonstrate the therapeutic benefit of mesenchymal stromal cell therapy in a premature piglet model of necrotizing enterocolitis. Improvements in clinical sickness scores, along with reductions in both gross and histologic intestinal injury, suggest that MSCS may play a critical role in preserving gut integrity under physiologic stress. The marked inflammatory response observed in NEC, and its attenuation following MSC treatment, further highlight the immunomodulatory potential of MSCs in this setting. This robust large-animal model lays the groundwork for future studies aimed at uncovering the molecular mechanisms underlying MSC-mediated protection in NEC.

## Supplementary Material

Supplementary Files

This is a list of supplementary files associated with this preprint. Click to download.

- SupplementaryTable1.tiff
- SupplementaryTable2.tif

## Figures and Tables

**Figure 1:**
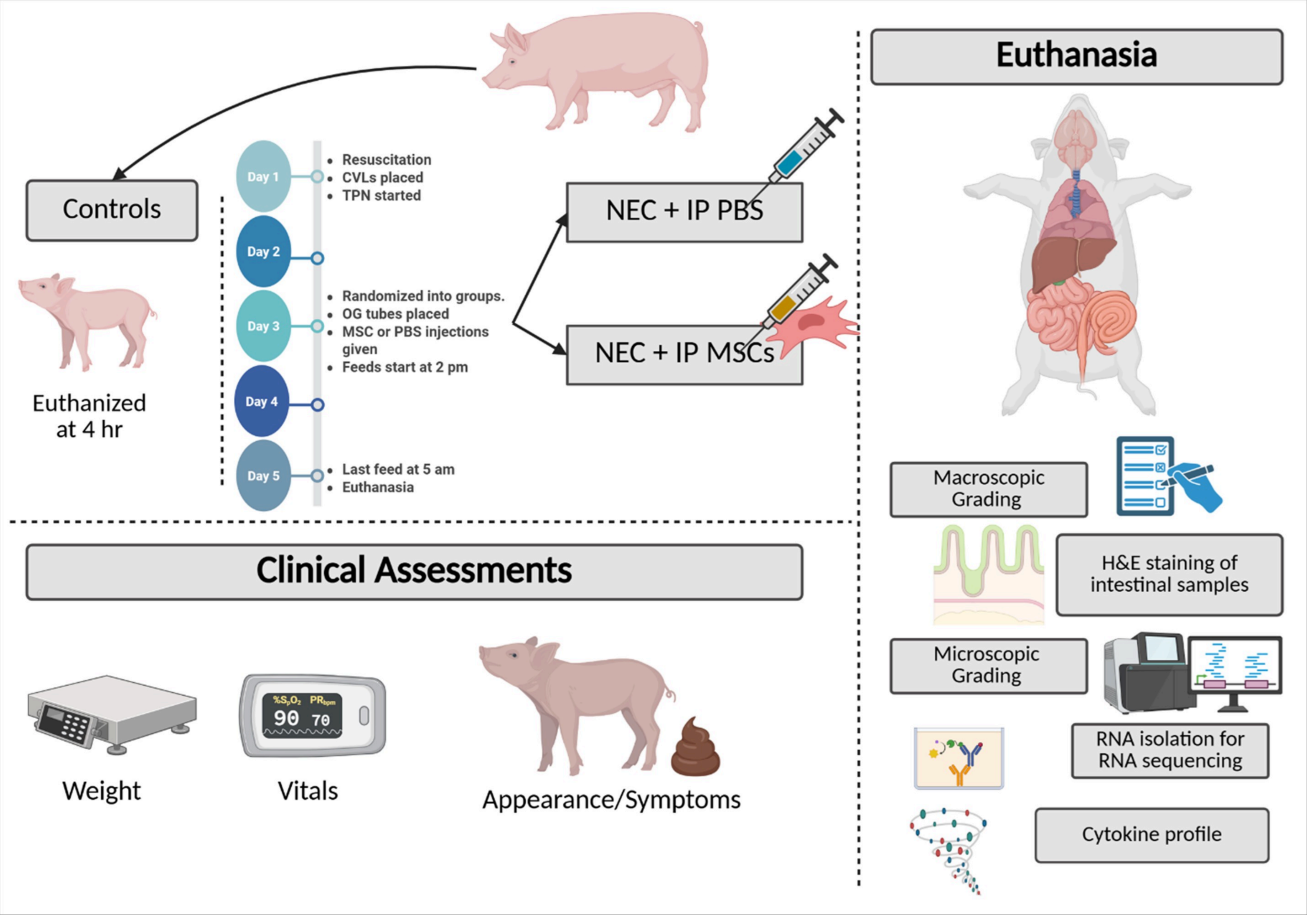
The Premature Piglet NEC Model. Schematic representation of the preterm piglet model of necrotizing enterocolitis (NEC) and treatment with intraperitoneal (IP) mesenchymal stromal cells (MSCs). Control piglets received resuscitation but were euthanized 4 hours after delivery without enteral feeding. Experimental piglets received central venous lines (CVLs) and total parenteral nutrition (TPN) on Day 1, were randomized to receive IP injections of either phosphate-buffered saline (PBS) or human MSCs on Day 3, and began enteral feeding on Day 3. Clinical assessments, including weight, vitals, and appearance/symptoms, were recorded daily. On Day 5, after the final feed, piglets were euthanized for sample collection. Post-mortem evaluations included gross and microscopic intestinal grading, hematoxylin and eosin (H&E) staining, cytokine profiling, and RNA sequencing of intestinal tissue. Created in https://BioRender.com

## Data Availability

The datasets generated and analyzed during the current study are available from the corresponding author upon reasonable request.

## References

[1] Fitzgibbons SC (2009) Mortality of necrotizing enterocolitis expressed by birth weight categories. J Pediatr Surg, 44(6): pp. 1072–5; discussion 1075–6 19524719 10.1016/j.jpedsurg.2009.02.013

[2] Moschino L (2021) Optimizing Nutritional Strategies to Prevent Necrotizing Enterocolitis and Growth Failure after Bowel Resection. Nutrients, 13(2) 10.3390/nu13020340 PMC7910892 33498880

[3] Meister AL, Doheny KK, Travagli RA (2020) Necrotizing enterocolitis: It’s not all in the gut. Exp Biol Med (Maywood) 245(2):85–95 31810384 10.1177/1535370219891971 PMC7016421

[4] Hunter CJ (2008) Understanding the susceptibility of the premature infant to necrotizing enterocolitis (NEC). Pediatr Res 63(2):117–123 18091350 10.1203/PDR.0b013e31815ed64c

[5] Neu J, Walker WA (2011) Necrotizing enterocolitis. N Engl J Med 364(3):255–264 21247316 10.1056/NEJMra1005408 PMC3628622

[6] Neu J (2014) Necrotizing enterocolitis: the mystery goes on. Neonatology 106(4):289–295 25171544 10.1159/000365130

[7] Neu J, Pammi M (2018) Necrotizing enterocolitis: The intestinal microbiome, metabolome and inflammatory mediators. Semin Fetal Neonatal Med 23(6):400–405 30172660 10.1016/j.siny.2018.08.001

[8] Lin PW, Stoll BJ (2006) Necrotising enterocolitis. Lancet 368(9543):1271–1283 17027734 10.1016/S0140-6736(06)69525-1

[9] Pierro A (1997) Necrotizing enterocolitis: pathogenesis and treatment. Br J Hosp Med 58(4):126–128 9373398

[10] Lin PW, Nasr TR, Stoll BJ (2008) Necrotizing enterocolitis: recent scientific advances in pathophysiology and prevention. Semin Perinatol 32(2):70–82 18346530 10.1053/j.semperi.2008.01.004

[11] Lengálová M (2022) Potential use of mesenchymal stem cells in the treatment of inflammatory bowel disease - a systematic review of the literature. Rozhl Chir 101(4):154–162 35623896 10.33699/PIS.2022.101.4.154-162

[12] Thébaud B (2019) Stem Cells for Extreme Prematurity. Am J Perinatol 36:02 10.1055/s-0039-1691774 31238363

[13] Pierro M, Thébaud B, Soll R (2017) Mesenchymal stem cells for the prevention and treatment of bronchopulmonary dysplasia in preterm infants. Cochrane Database Syst Rev, 11(11): p. Cd011932. 29125893 10.1002/14651858.CD011932.pub2 PMC6485972

[14] Augustine S (2017) Mesenchymal Stromal Cell Therapy in Bronchopulmonary Dysplasia: Systematic Review and Meta-Analysis of Preclinical Studies. Stem Cells Transl Med 6(12):2079–2093 29045045 10.1002/sctm.17-0126 PMC5702524

[15] Ma QQ (2019) Bone marrow mesenchymal stem cells modified by angiogenin-1 promotes tissue repair in mice with oxygen-induced retinopathy of prematurity by promoting retinal stem cell proliferation and differentiation. J Cell Physiol 234(11):21027–21038 31106420 10.1002/jcp.28706

[16] McCulloh CJ (2017) Stem cells and necrotizing enterocolitis: A direct comparison of the efficacy of multiple types of stem cells. J Pediatr Surg 52(6):999–1005 28366560 10.1016/j.jpedsurg.2017.03.028 PMC5467690

[17] Wei J, Zhou Y, Besner GE (2015) Heparin-binding EGF-like growth factor and enteric neural stem cell transplantation in the prevention of experimental necrotizing enterocolitis in mice. Pediatr Res 78(1):29–37 25806717 10.1038/pr.2015.63 PMC4472527

[18] Yang J (2012) Heparin-binding epidermal growth factor-like growth factor and mesenchymal stem cells act synergistically to prevent experimental necrotizing enterocolitis. J Am Coll Surg 215(4):534–545 22819639 10.1016/j.jamcollsurg.2012.05.037 PMC3444529

[19] Zhou Y (2013) Enteric nervous system abnormalities are present in human necrotizing enterocolitis: potential neurotransplantation therapy. Stem Cell Res Ther 4(6):157 24423414 10.1186/scrt387 PMC4054965

[20] Tayman C (2011) Mesenchymal stem cell therapy in necrotizing enterocolitis: a rat study. Pediatr Res 70(5):489–494 21772224 10.1203/PDR.0b013e31822d7ef2

[21] Drucker NA (2019) Inhibiting hydrogen sulfide production in umbilical stem cells reduces their protective effects during experimental necrotizing enterocolitis. J Pediatr Surg 54(6):1168–1173 30879750 10.1016/j.jpedsurg.2019.02.037 PMC6545254

[22] Te Winkel J (2019) Mesenchymal stem cells promote mesenteric vasodilation through hydrogen sulfide and endothelial nitric oxide. Am J Physiol Gastrointest Liver Physiol 317(4):G441–g446 31343254 10.1152/ajpgi.00132.2019 PMC6842994

[23] Jensen AR (2018) Umbilical mesenchymal stromal cells provide intestinal protection through nitric oxide dependent pathways. J Surg Res 224:148–155 29506832 10.1016/j.jss.2017.11.068 PMC6128694

[24] Yin L (2017) Pig models on intestinal development and therapeutics. Amino Acids 49(12):2099–2106 28986749 10.1007/s00726-017-2497-z

[25] Guilloteau P (2010) Nutritional programming of gastrointestinal tract development. Is the pig a good model for man? Nutr Res Rev 23(1):4–22 20500926 10.1017/S0954422410000077

[26] Robinson JL (2018) Prematurity reduces citrulline-arginine-nitric oxide production and precedes the onset of necrotizing enterocolitis in piglets. Am J Physiol Gastrointest Liver Physiol 315(4):G638–g649 30048597 10.1152/ajpgi.00198.2018 PMC6415715

[27] Sangild PT (2006) Diet- and Colonization-Dependent Intestinal Dysfunction Predisposes to Necrotizing Enterocolitis in Preterm Pigs. Gastroenterology 130(6):1776–1792 16697741 10.1053/j.gastro.2006.02.026

[28] Ghoneim N (2014) Delayed Initiation but Not Gradual Advancement of Enteral Formula Feeding Reduces the Incidence of Necrotizing Enterocolitis (NEC) in Preterm Pigs. PLoS ONE 9(9):e106888 25238061 10.1371/journal.pone.0106888 PMC4169518

[29] Stoll B (2012) Continuous parenteral and enteral nutrition induces metabolic dysfunction in neonatal pigs. JPEN J Parenter Enter Nutr 36(5):538–550 10.1177/0148607112444756 PMC4000127 22549765

[30] Zamora IJ (2015) Low Abdominal NIRS Values and Elevated Plasma Intestinal Fatty Acid-Binding Protein in a Premature Piglet Model of Necrotizing Enterocolitis. PLoS ONE 10(6):e0125437 26061399 10.1371/journal.pone.0125437 PMC4465330

[31] Ragan MV (2023) Development of a novel definitive scoring system for an enteral feed-only model of necrotizing enterocolitis in piglets. Front Pediatr 11:1126552 37138566 10.3389/fped.2023.1126552 PMC10149862

[32] Dobin A (2013) STAR: ultrafast universal RNA-seq aligner. Bioinformatics 29(1):15–21 23104886 10.1093/bioinformatics/bts635 PMC3530905

[33] Breese MR, Liu Y (2013) NGSUtils: a software suite for analyzing and manipulating next-generation sequencing datasets. Bioinformatics 29(4):494–496 23314324 10.1093/bioinformatics/bts731 PMC3570212

[34] Liao Y, Smyth GK, Shi W (2014) featureCounts: an efficient general purpose program for assigning sequence reads to genomic features. Bioinformatics 30(7):923–930 24227677 10.1093/bioinformatics/btt656

[35] Robinson MD, McCarthy DJ, Smyth GK (2010) edgeR: a Bioconductor package for differential expression analysis of digital gene expression data. Bioinformatics 26(1):139–140 19910308 10.1093/bioinformatics/btp616 PMC2796818

[36] Gephart SM (2012) Necrotizing enterocolitis risk: state of the science. Adv Neonatal Care 12(2):77–87 quiz 88 – 9 22469959 10.1097/ANC.0b013e31824cee94 PMC3357630

[37] Jiang H (2011) Bone marrow mesenchymal stem cells reduce intestinal ischemia/reperfusion injuries in rats. J Surg Res 168(1):127–134 19932900 10.1016/j.jss.2009.07.035

[38] Markel TA (2020) Human Mesenchymal Stem Cell Hydrogen Sulfide Production Critically Impacts the Release of Other Paracrine Mediators After Injury. J Surg Res 254:75–82 32417499 10.1016/j.jss.2020.04.014 PMC7483380

[39] Crisostomo PR (2008) Human mesenchymal stem cells stimulated by TNF-alpha, LPS, or hypoxia produce growth factors by an NF kappa B- but not JNK-dependent mechanism. Am J Physiol Cell Physiol 294(3):C675–C682 18234850 10.1152/ajpcell.00437.2007

[40] Weil BR (2009) Mesenchymal stem cells enhance the viability and proliferation of human fetal intestinal epithelial cells following hypoxic injury via paracrine mechanisms. Surgery 146(2):190–197 19628073 10.1016/j.surg.2009.03.031

[41] Liau LL (2020) The Potential of Mesenchymal Stromal Cell as Therapy in Neonatal Diseases. Front Pediatr 8:591693 33251167 10.3389/fped.2020.591693 PMC7672022

[42] Jensen AR (2017) Hydrogen sulfide improves intestinal recovery following ischemia by endothelial nitric oxide-dependent mechanisms. Am J Physiol Gastrointest Liver Physiol 312(5):G450–g456 28280145 10.1152/ajpgi.00444.2016 PMC5451562

[43] Jensen AR (2017) Hydrogen Sulfide: A Potential Novel Therapy for the Treatment of Ischemia. Shock 48(5):511–524 28498298 10.1097/SHK.0000000000000894

[44] Jensen AR (2020) Stem Cell Therapy and Hydrogen Sulfide: Conventional or Nonconventional Mechanisms of Action? Shock 53(6):737–743 31348146 10.1097/SHK.0000000000001420 PMC6980464

[45] Drucker NA (2018) Hydrogen sulfide provides intestinal protection during a murine model of experimental necrotizing enterocolitis. J Pediatr Surg 53(9):1692–1698 29338840 10.1016/j.jpedsurg.2017.12.003

[46] Hunter CE (2023) Hydrogen Sulfide Improves Outcomes in a Murine Model of Necrotizing Enterocolitis via the Cys440 Residue on Endothelial Nitric Oxide Synthase. J Pediatr Surg 58(12):2391–2398 37684170 10.1016/j.jpedsurg.2023.08.006 PMC10841167

[47] Fiarresga A (2015) Intracoronary Delivery of Human Mesenchymal/Stromal Stem Cells: Insights from Coronary Microcirculation Invasive Assessment in a Swine Model. PLoS ONE 10(10):e0139870 26479722 10.1371/journal.pone.0139870 PMC4610677

[48] Grieve SM (2010) Microvascular obstruction by intracoronary delivery of mesenchymal stem cells and quantification of resulting myocardial infarction by cardiac magnetic resonance. Circ Heart Fail 3(3):e5–6 20484192 10.1161/CIRCHEARTFAILURE.109.931360

[49] Coppin L, Sokal E, Stéphenne X (2019) Thrombogenic Risk Induc Intravascular Mesenchymal Stem Cell Therapy: Curr Status Future Perspect Cells, 8(10) 10.3390/cells8101160 PMC6829440 31569696

[50] Li K (2023) Dose-specific efficacy of adipose-derived mesenchymal stem cells in septic mice. Stem Cell Res Ther 14(1):32 36804962 10.1186/s13287-023-03253-3 PMC9940377

[51] Villamor-Martinez E (2020) Stem Cells as Therapy for Necrotizing Enterocolitis: A Systematic Review and Meta-Analysis of Preclinical Studies. Front Pediatr 8:578984 33363060 10.3389/fped.2020.578984 PMC7755993

[52] Nitkin CR (2020) Stem cell therapy for preventing neonatal diseases in the 21st century: Current understanding and challenges. Pediatr Res 87(2):265–276 31086355 10.1038/s41390-019-0425-5 PMC6854309

